# Supplementary material for: A nanoscale robotic cleaner
Source: Nat Commun. 2026 Mar 27;17:3027. doi: 10.1038/s41467-026-70685-9 (PMC13035946; doi:10.1038/s41467-026-70685-9)
Supplement: Supplementary file 1 — Supplementary Information [file 41467_2026_70685_MOESM1_ESM.pdf]

Supplementary Information for
A nanoscale robotic cleaner

Jin Qin[1*], Carsten Büchner[1], Xiaofei Wu[2], Bert Hecht[1*]

[1]Nano-Optics and Biophotonics Group, Experimentelle Physik 5, Physikalisches Institut, Universität Würzburg, Am Hubland, Würzburg, D-97074, Germany.
[2]Leibniz Institute of Photonic Technology, Albert-Einstein-Straße 9, Jena, D-07745, Germany.

*Corresponding author(s). E-mail(s): jin.qin@uni-wuerzburg.de; bert.hecht@uni-wuerzburg.de;

Contents

S1 Optical setup **3**

S2 Comparison with drone-like and car-like designs **4**

S3 Image processing **4**

S4 Plasmonic motors optimizations **5**

S5 Orientational trapping and steering **7**

S6 Comparison of two different orientation control modes **8**
S6.1 Rotating a linear polarizer 8
S6.2 Applying a short pulse of circularly polarized laser 10

S7 Bacteria trapping behaviour **11**
S7.1 Comparison of optical and thermophoretic forces for bacterial trapping 11
S7.2 3D thermophoretic trapping 12
S7.3 Hydrodynamic drag force on trapped bacteria 12

S8 Maneuverability of robots under bacterial loading **13**

S8.1 Maximum speed after bacteria assembly 13
S8.2 Turning precision after bacteria assembly 14
S8.3 Cleaning efficiency over a static or mobile robots 15
S8.4 Theoretical estimation of effective Soret Coefficient 15

S9 Motion dynamics **17**
S9.1 Out-of-plane stability 17
S9.2 In-plane motion dynamics 17

S10 Temperature distribution and thermo-induced convective flow **19**

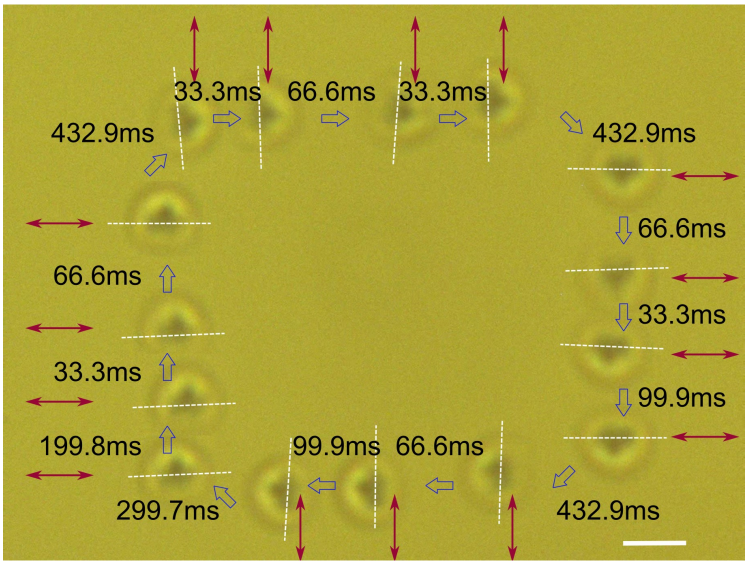

Fig. S1 Time intervals between the superimposed frames in Fig. 1b.

S1 Optical setup

Laser light emitted from a laser diode (980 nm, Thorlabs, BL976-PAG900) first passes through a linear polarizer and a quarter-wave plate, converting it into circularly polarized light. The beam is then aligned to match the aperture of the electro-optic (EO) modulator (Thorlabs, EO-AM-NR-C2). By applying different voltages to the birefringent lithium niobate crystal, phase differences are induced along its two orthogonal axes, effectively altering the laser's polarization. Two EO modulators are employed, each operating under different voltage conditions. For instance, when voltages of $\pm V_{\pi/4}$ is applied, corresponding to a phase shift of $\pm\pi/4$, the initially circularly polarized light is converted into vertical (VP) or horizontal (HP) linear polarization. The corresponding voltages for different polarization states are summarized in Table S1. The laser power can be tuned by adjusting the applied voltages between -10V (minimum) and 10V (maximum). In combination with an additional polarizer, the output laser power can be modulated by adjusting the applied voltage, as illustrated in Fig. S2. The laser spot used in the experiments has a $1/e^2$ diameter of 20 µm, defined as the distance at which the intensity drops to $1/e^2$ of the peak value, determined by fitting the spot profile with a 2D Gaussian function.

Table S1 Applied EOM voltages used to produce the corresponding phase shifts.

Applied voltage	-10V	0V	10V
Phase shift	$-\pi/4$	0	$\pi/4$

S2 Comparison with drone-like and car-like designs

In the drone-like system, we focus more on higher maneuverability, achieved via circularly polarized light fields with opposite helicities which generate counteracting optical torques to balance orientation during propulsion. In contrast, the car-like design focuses more on high propulsion efficiency. It employs a single plasmonic directional antenna illuminated by a linearly polarized light field. This configuration provides both unidirectional photon-recoil thrust and a passive torque trap that aligns the robot's orientation with respect to the polarization axis, eliminating the need for multi-beam torque balancing. Below, we summarize the key advantages and trade-offs of both approaches in Table. S2.

Table S2 Comparison between drone-like and car-like designs.

Feature	Drone-like Design	This Work: Car-like design
Typical Size (Diameter)	2.5 μm	920nm (nanorobot)<br>1.5μm (microrobot)
Propulsion Mechanism	Photon recoil from chiral plasmonic antennas	Photon recoil from directional plasmonic antenna+ Orientation locking
Control Inputs	Two independent circularly polarized beams	One unfocused beam; polarization sequencing (linear + circular)
Degrees of Freedom	All degrees of freedom in 2D translation + rotation	Translation with polarization-locked orientation (rotation direction controlled via CP pulses)
Propulsion Efficiency (velocity per optical power)	13.3 $\frac{\mu m/s}{mW/\mu m^2}$	46.9 $\frac{\mu m/s}{mW/\mu m^2}$ (microrobot)<br>121.2 $\frac{\mu m/s}{mW/\mu m^2}$ (nanorobot)
Typical Speed	8 $\mu m/s$	15 $\mu m/s$ (microrobot)<br>40 $\mu m/s$(nanorobot)
Optical Setup	Requires beam splitting, alignment, and power balancing	Single beam
Primary Advantage	Full maneuverability in 2D plane	Miniaturization + enhanced propulsion efficiency + simplified setup
Primary Trade-off	More complex setup	Reduced degrees of freedom (addressed via polarization sequencing)

S3 Image processing

To track the real-time positions of micro- and nanorobots, the open-source OpenCV library (Python) is used to extract the coordinates of plasmonic motors by identifying black spots in the frames. First, the colorful frame is binarized by applying a threshold based on the intensity of the target of interest. Then, an erosion operation is performed to eliminate small noise artifacts. Next, connected domains are identified, and their respective mass centers are extracted. The position of the microrobot is determined by averaging the coordinates of all four extracted mass centers which

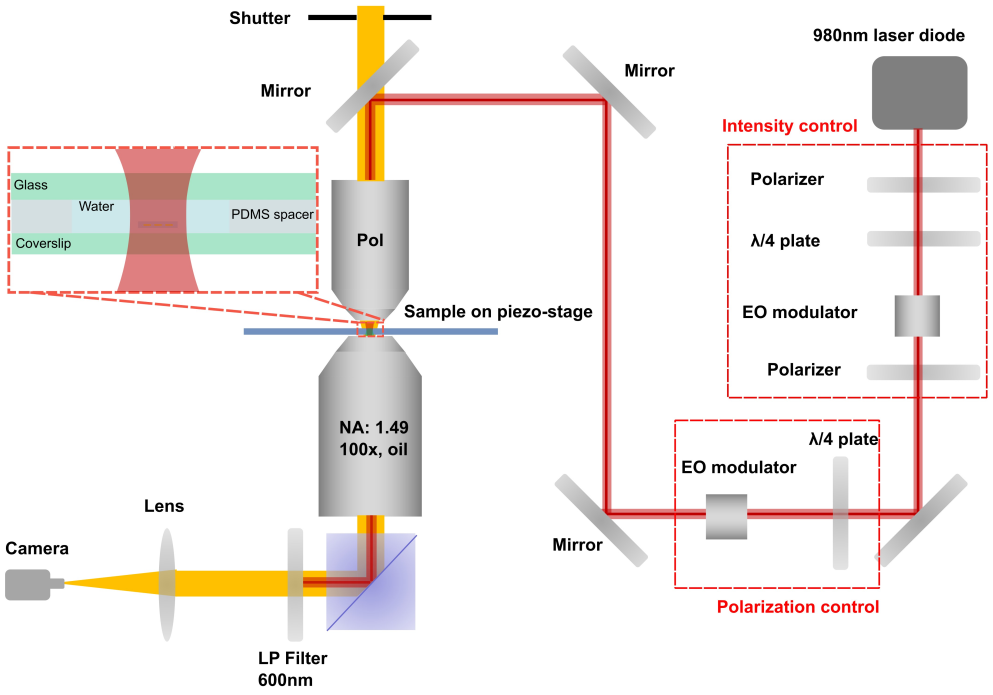

Fig. S2 The optical setup for micro- and nanorobot manipulation. Two dashed rectangular boxes indicate the laser control and polarization control sections, respectively. The left-top inset indicates the zoom image of the liquid cell contains a microrobot.

correspond to the 4 pairs of gold nanorods. Additionally, the orientation of the microrobot can be inferred from the four detected spots. This is based on the difference in spacing between opposing pairs of plasmonic dimers and self-oriented nanorods in the microrobot design, where the former exhibits a slightly larger separation. Using this criterion, the orientation angle of the microrobot is determined by analyzing the angle of the connection line along the long axis. A similar approach can be applied to nanorobot tracking for real-time position analysis. However, due to image resolution limitations, extracting the orientation of nanorobots is challenging, as their plasmonic motors typically appear as a single black spot in the images.

S4 Plasmonic motors optimizations

The plasmonic dimer for unidirectional scattering is optimized by varying the lengths of the two nanorods and their spacing. According to the Yagi-Uda design principle, the two nanorods should exhibit differing but overlapping dipolar radiative resonances which can effectively be excited at the same wavelength but lead to oscillations with a certain phase shift. Additionally, the spacing distance induces an extra phase shift, leading to constructive interference in one direction perpendicular to the rod's long

axes and destructive interference in the opposite direction. After several iterations, as shown in Fig. S3a, a double-resonance feature appears in the scattering cross-section, indicating resonances at nanorod lengths of 170 nm and 150 nm, respectively. Addressing the plasmonic dimer at the dip position maximizes the optical force since both resonances are excited with the same amplitude. The resulting optimal spacing distance is 210 nm.

The geometrical anisotropy of the structure induces a self-correcting optical torque when the nanorod's long axes are misaligned with the direction of linear polarization [1, 2]. The optical torque induced by linearly polarized light can be modeled as follows. Considering a plane wave excitation $\mathbf{E} = \mathbf{E}_0 \cos(\omega t)$, the nanorod exhibits a typical dipolar mode under linear polarization. The induced electric dipole moment is given by $\mathbf{p} = \bar{\bar{\alpha}}\mathbf{E}$, where $\bar{\bar{\alpha}}$ is the polarizability tensor. In a laboratory frame where the x-axis aligns with the nanorod's major axis, the polarizability tensor takes the diagonal form:

$$\bar{\bar{\alpha}} = \begin{bmatrix} \alpha_{\parallel} & 0 & 0 \\ 0 & \alpha_{\perp} & 0 \\ 0 & 0 & \alpha_{\perp} \end{bmatrix}. \tag{1}$$

The incident electric field in this laboratory frame can be expressed as:

$$\mathbf{E} = E_0 \cos(\omega t)[\cos(\varphi)\hat{\mathbf{x}} + \sin(\varphi)\hat{\mathbf{y}}]. \tag{2}$$

The torque $\mathbf{T}$ acting on the dipole in an electric field is given by:

$$\begin{aligned} \mathbf{T} &= \mathbf{p} \times \mathbf{E} \\ &= E_0^2 \cos^2(\omega t) \begin{vmatrix} \hat{\mathbf{x}} & \hat{\mathbf{y}} & \hat{\mathbf{z}} \\ \alpha_{\parallel} \cos(\varphi) & \alpha_{\perp} \sin(\varphi) & 0 \\ \cos(\varphi) & \sin(\varphi) & 0 \end{vmatrix} \\ &= {E_0}^2 (\alpha_{\parallel} - \alpha_{\perp}) \cos(\varphi) \sin(\varphi) \cos^2(\omega t)\hat{\mathbf{z}} \\ &= \frac{1}{2}{E_0}^2 (\alpha_{\parallel} - \alpha_{\perp}) \sin(2\varphi) \cos^2(\omega t)\hat{\mathbf{z}}. \end{aligned} \tag{3}$$

Taking the time average, $\langle \cos^2(\omega t) \rangle = \frac{1}{2}$, the time-averaged torque becomes:

$$\langle \mathbf{T} \rangle = \frac{1}{4}{E_0}^2 \operatorname{Re}(\alpha_{\parallel} - \alpha_{\perp}) \sin(2\varphi)\hat{\mathbf{z}}. \tag{4}$$

From Eq. 4 we see that the optical torque induced by linearly polarized light follows a typical $\sin(2\varphi)$ dependence and reaches a maximum at $\varphi = 45°$. Additionally, the torque is proportional to the real part of the polarizability. Notably, when the dipolar mode of the nanorod resonates with the incident electric field, the induced optical torque becomes zero due to the additional $\pi/2$ phase shift between the incident field and the induced dipolar response.

As shown in Fig. S3b, simulations reveal that the optical torque of a nanorod (Length: 150 nm, Width: 60 nm) embedded in a matrix of HSQ varies under linear (LP) and circular polarization (CP). The nanorod exhibits a resonance at 920 nm, where the optical torque induced by spin momentum transfer is maximized, while the self-induced torque under LP excitation is zero. However, at 980 nm, the LP-induced

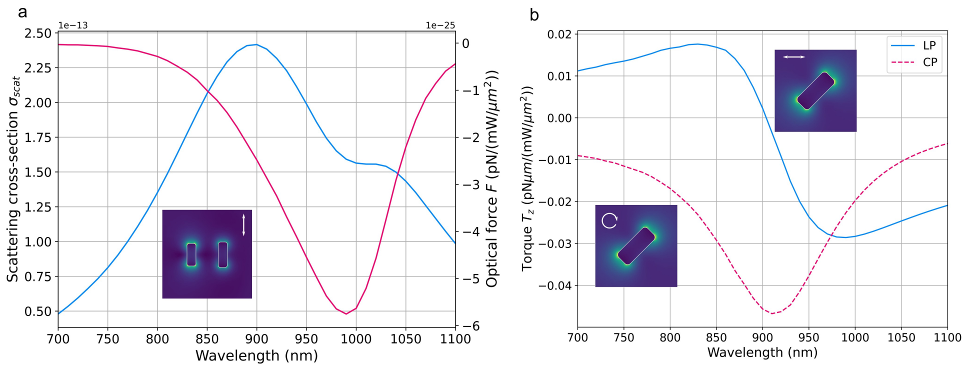

Fig. S3 Plasmonic Structure Optimization. a, Scattering cross-section and corresponding time-averaged optical force of the plasmonic dimer. The inset illustrates the electric field distribution when the structure is resonantly excited. **b**, Self-correcting torque on a single nanorod (blue solid line) under LP and the corresponding torque under CP laser excitation. The inset depicts the simulation configurations.

torque reaches its peak. Interestingly, this self-orienting torque can be both positive and negative, indicating that the nanorod can align either parallel or perpendicular to the linear polarization.

To optimize plasmonic motor structures for micro- and nanorobots, two key factors should be considered: maximizing the optical thrust force at an orientation angle of 0° and maximizing the self-correcting optical torque at 45°. In microrobot configurations, self-correcting motors consist of four nanorods arranged to minimize their impact on the optical thrust force. In nanorobot designs, the plasmonic dimer antenna is surrounded by multiple nanorods optimized for self-correction effects. Further optimization can be achieved using computational algorithms to iteratively refine the structure. The larger number of nanorods responsible for the orientational trapping leads to a more dipole like emission pattern which is superimposed to the directional scattering of the Yagi-Uda core elements. The detailed geometries of the two robotic devices are shown in Fig. S4.

S5 Orientational trapping and steering

In our experiments the robotic device is steered along a rectangular trajectory. At each turn, a short pulse of circular polarization is applied to slightly reorient the device by means of spin momentum transfer. Only afterwards the linear polarization is switched and the robotic device fully reorients into the new equilibrium orientation. To more accurately evaluate the orientational trap capability (standard deviation of the reduced orientation angle θ'), we consider the straight path segments by only considering the data where $|\theta'| < 15°$, as indicated in Fig. S5a. Under this criterion, a standard deviation of ±6.7° is extracted when the laser intensity is $0.24\,\mathrm{mW/\mu m^2}$, as shown in Fig. S5c. For comparison, a Gaussian fit applied to the data (Fig. S5d)

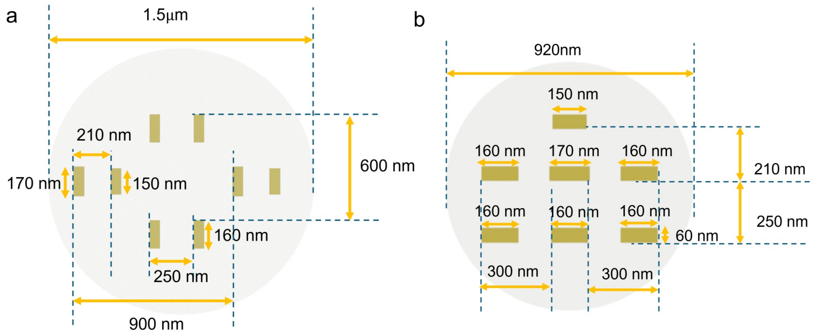

Fig. S4 Detailed geometric parameters of the microrobot (a) and nanorobot (b).

when the laser intensity is $0.38\,\mathrm{mW/\mu m^2}$, which yielding a slightly narrower standard deviation of $\pm 6.3°$.

Furthermore, the depth of orientational trapping potential is expected to increase with higher laser intensities, as a deeper effective trapping potential reduces thermal orientation fluctuations inside a orientational trap. This is confirmed in Fig. S5b, where the standard deviation of θ' decreases with increasing laser intensity, indicating stronger orientation stabilization.

S6 Comparison of two different orientation control modes

We now present a theoretical model that distinguishes between two control modes: smooth trajectory steering using slowly rotating linear polarization, and deterministic rapid turning enabled by brief pulses of circularly polarized light.

S6.1 Rotating a linear polarizer

To analyze the ability of the robot to follow a rotating polarization state, we consider the optical torque generated by the self-alignment mechanism. For the microrobot (Fig. 2), the optical torque can be expressed as:

$$\tau_{opt} = -\tau_0 \sin(2\theta) \tag{5}$$

where θ is the robot's orientation relative to the linear polarization. When the polarization direction is rotated at a constant angular speed ω, the robot experiences both this optical restoring torque and a viscous drag torque. In the low-Reynolds-number regime, the drag torque acting on the microrobot increases linearly with the rotational velocity ω_r, yielding: $\tau_{drag} = k\omega_r$, where the rotational drag coefficient k is obtained from CFD simulations. For the robot geometry and a robot–substrate spacing of 500

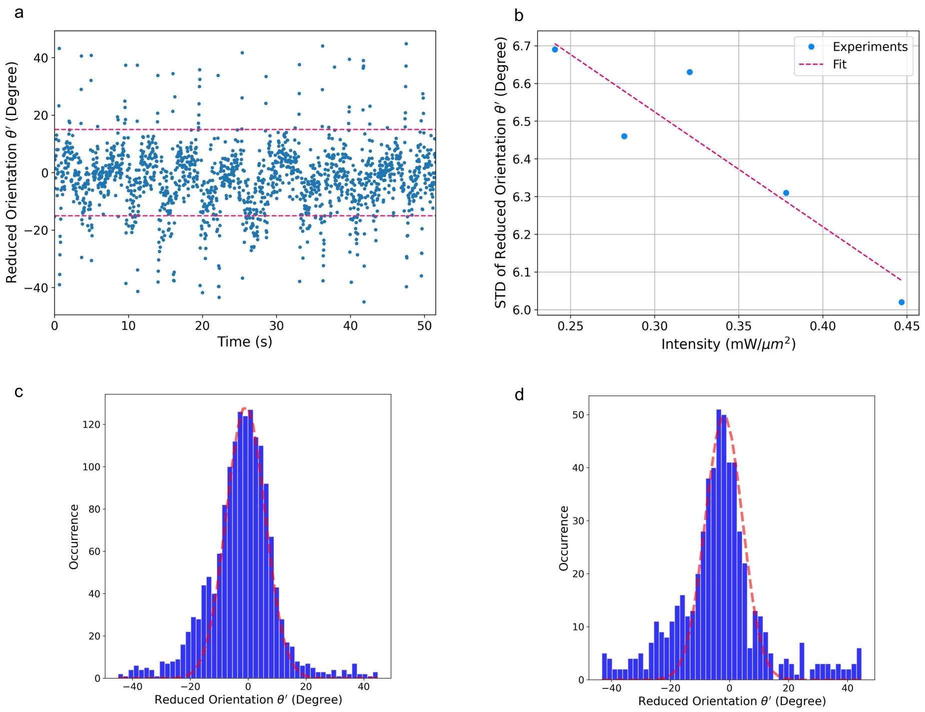

Fig. S5 Orientation trap capability. **a**, Time-dependent reduced orientation angle θ' when the laser intensity is $0.24\,\mathrm{mW/\mu m^2}$. The pink dashed lines ($\pm15°$) indicate the range considered for Gaussian fitting in **c**. **b**, Standard deviation of θ' under different laser intensities, showing a linearly decreasing trend with increasing intensity. **c**, Gaussian fitting with a standard deviation of $\pm6.7°$ applied to the histogram in **a**. **d**, Gaussian fitting with a standard deviation of $\pm6.3°$ when the laser intensity is $0.38\,\mathrm{mW/\mu m^2}$.

nm, the simulated relationship between drag torque and angular velocity is shown in Fig. S13c. Transforming to the frame rotating with the polarization angle $\phi(t) = \omega t$, the relative angle θ obeys:

$$\theta = \omega t - \int_0^t \omega_r d\omega_r \tag{6}$$

Balancing optical torque with drag torque leads to the rotational dynamics:

$$\dot{\theta} = \omega - \tau_0/k \sin(2\theta) \tag{7}$$

In a steady state ($\dot{\theta} = 0$), we obtain: $\sin(2\theta^*) = \omega/(\tau_0/k) = \omega/\Omega$

A real, stable solution exists only when $\omega < \Omega$. In this regime, the robot maintains a constant phase lag relative to the rotating polarization and follows the linear polarization smoothly. When $\omega > \Omega$, no steady-state solution exists, and the orientation angle continually drifts—i.e., the robot cannot keep up with the polarization rotation and exhibits slip. Figures S6a and S6b show numerical solutions in both regimes. The

locking rate Ω can be estimated to be 7.97 rad/s at a laser intensity of $0.3\ mW/\mu m^2$. At a moderate rotation rate (e.g., $\omega = 2rad/s$, the robot tracks the polarization closely, whereas at higher rotation speeds the orientation fails to follow and slips repeatedly. In this simplified model, we neglect Brownian rotational noise. However, this noise becomes important during rapid 90° polarization changes: when the optical potential is at metastable position, thermal fluctuations can push the robot into a stable region, causing the turning direction (left vs. right) to become nondeterministic.

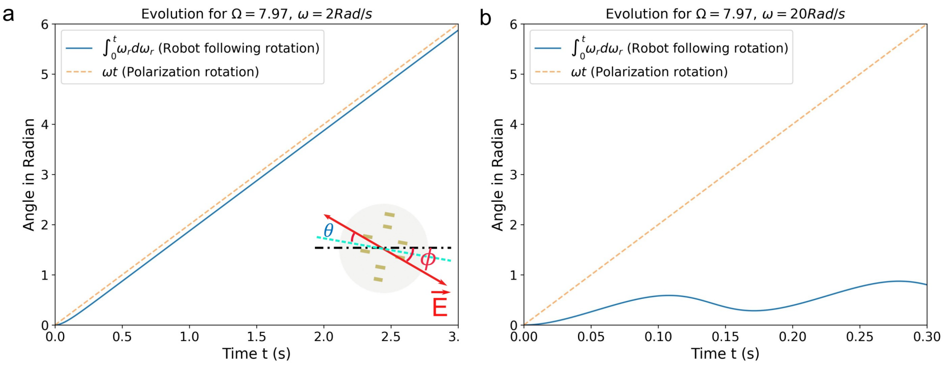

Fig. S6 Numerical solutions of the rotational dynamics. a, Stable phase locking occurs when the polarization rotation rate ω is smaller than the locking rate Ω. *Inset:* Schematic of the simulation model. **b**, Time evolution of the polarization angle and the corresponding robot orientation for $\omega > \Omega$, showing that the robot cannot faithfully track the rotating polarization.

S6.2 Applying a short pulse of circularly polarized laser

When a short circularly polarized light pulse is applied, the robot experiences a unidirectional optical torque, resulting in deterministic rotation at an angular velocity of approximately 11.8 rad/s (as derived from Fig. 2b). A pulse duration of ∼100 ms therefore produces a rotation of about 67.7° in the ideal, noise-free case—sufficient to reliably nudge the robot to rotate into the correct direction. From the analysis above, both methods can be used to tune the robot's orientation. However, applying short pulses of circularly polarized light is more suitable for rapid reorientation necessary under high-speed opertion. As discussed earlier, the rotation speed of a linear polarizer is inherently limited; if it is too fast, the device's orientation cannot follow, leading to orientation slip. This consideration underlies our demonstration design: because the robot operates at relatively high velocities, prompt and reliable orientation changes are essential. Besides, rapid polarization switching combined with spin angular momentum transfer provides a deterministic way of executing sharp turns, thereby avoiding large-radius curves and enabling precise cleaning operations.

As discussed above, continuous rotation of the linear polarization would allow smooth trajectory control entirely through the self-alignment mechanism, provided

the rotation speed remains below the locking rate Ω. This capability could be realized in future experiments by integrating a motorized rotating polarizer for continuous polarization rotation.

S7 Bacteria trapping behaviour

S7.1 Comparison of optical and thermophoretic forces for bacterial trapping

In Fig. S7, we provide a direct comparison between the simulated optical forces and the thermophoretic forces acting on bacteria near the robot. For simplicity, we model each bacterium as a sphere of 1 μm diameter with a refractive index of 1.38 [3]. The optical trapping force on each bacterium is obtained by integrating Maxwell's stress tensor over its surface. As shown in Fig. S7a, the optical force is on the order of a few femtonewtons at an excitation intensity of 0.3 $mW/\mu m^2$, and its direction does not consistently point toward the robot center. This behavior arises because the plasmonic motors are designed to scatter light asymmetrically to generate photon recoil, resulting in a highly nonuniform near-field around the robot; combined with the fact that the bacterial refractive index is close to that of water, the optical gradient force is far too weak to drive cluster formation.

In contrast, the thermophoretic force, originating from the temperature gradient around the robot, always points from the colder fluid toward the hotter region near the plasmonic motor. As shown in Fig. S7b, its magnitude is orders of magnitude larger than the optical gradient force, and it consistently directs bacteria toward the robot center, explaining the observed long-range and robust assembly behavior.

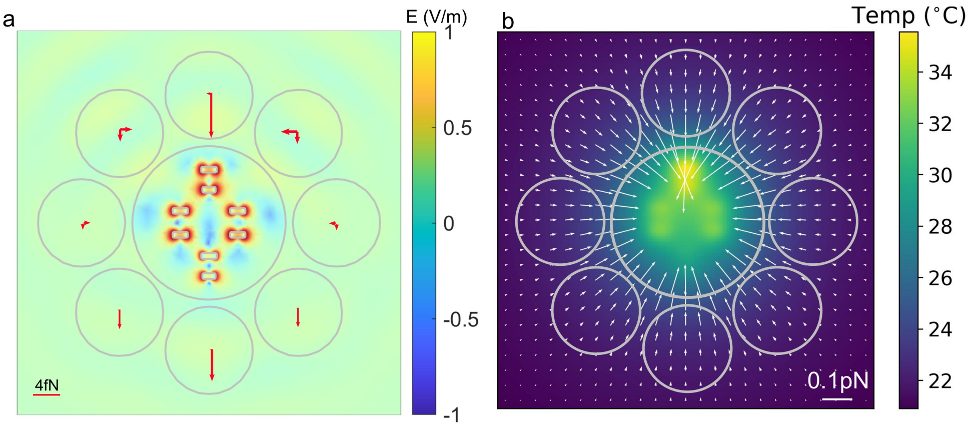

Fig. S7 Comparison of optical and thermophoretic forces acting on bacteria near the robot. a Near field electric-field distribution (logarithmic scale) under x-polarized excitation, with red arrows indicating the optical force vectors (both directions and amplitudes) on individual bacteria. **b** Temperature distribution under the same illumination, with white arrows indicating the magnitude and direction of the resulting thermophoretic forces.

S7.2 3D thermophoretic trapping

Similar as the thermophoretic trapping potential in the xy-plane shown in Fig. 4d, the temperature gradient in xz plane also can contribute to a effective trapping potential. The temperature distribution in xz plane was simulated in Fig. S8a. Using the Soret coefficient estimated from our measurements, we calculated the corresponding thermophoretic potential landscape (Fig. S8b). This analysis shows that trapping is not restricted to the 2D xy-plane but extends into the full 3D space surrounding the robot. In particular, the thermophoretic force exceeds the hydrodynamic drag over a broad region above and around the robot, pulling approaching bacteria toward the device from all directions. The resulting equipotential surfaces form a semi-hemispherical confinement region, consistent with a true 3D trapping volume. Due to limitations of optical imaging, bacteria trapped directly above the microrobot or nanorobot cannot always be clearly resolved. Nonetheless, this behavior is observed experimentally: during the collection process, not all bacteria accumulate solely at the rim of the device—some are captured and stacked on the top surface, confirming the presence of out-of-plane thermophoretic confinement.

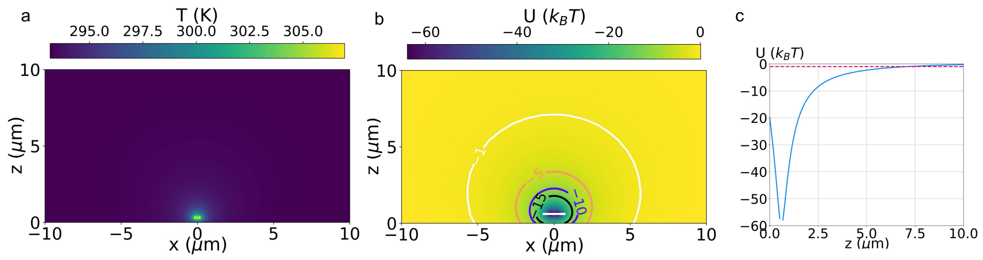

Fig. S8 Thermophoretic trapping behaviour in xz plane. a, temperature distribution around the four-motor design when averaged laser poser is 0.28 $mW/\mu m^2$. **b**, The calculated Thermophoretic trapping potential with assigned Soret coefficients of 2.45 K^{-1}. **c**, A linecut of trapping potential in b when x=0.

S7.3 Hydrodynamic drag force on trapped bacteria

When a bacterium-loaded robot moves at a finite velocity, the hydrodynamic drag acting on the attached bacteria can challenge effective trapping. To assess the influence of this drag force, we develop a model to quantitatively analyze the trapping stability. Taking carnosus bacteria (diameter of roughly $1\mu m$) as examples, the hydrodynamic drag force, which acting on the bacterium carried by a moving microrobot, can be expressed as $F_d = 6\pi\eta r v$, where η is water viscosity, r, v are the radius and velocity of moving bacterium respectively. As we see in Supplementary Video 5, when a large bunch of bacteria is assembled around the microrobot, the typical moving velocity is

quite low (below $0.5\mu m/s$). In this case, the hydrodynamic drag force can be estimated as 4.2 fN, which is far below the thermophoretic trapping force, which is estimated by $F_t = k_B T S_T \nabla T$. Taking the value of $\nabla T = 10^7 K/m$, F_t is 0.1 pN, which is roughly 20 times larger than the drag force. But the existence of this drag force still will distort the trapping potential as shown in Fig. S9, where we tune the moving velocity from 0 nm/s to 500 nm/s. As we can see from the inset, when the microrobot moves left with trapped bacteria, for the left-most trapped bacterium, the drag force effectively will enhance the trap performance. In the contrary, for the right-most bacterium, the drag force will counteract the thermophoretic force, leading to a decrease of the effective trapping potential.

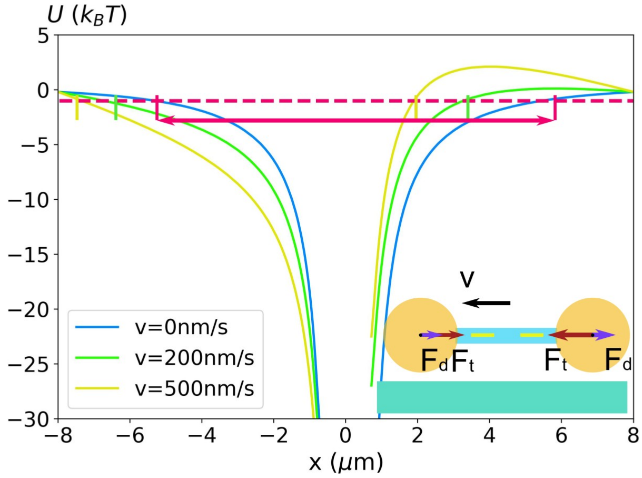

Fig. S9 Illustration of trapping potential with the influence of hydrodynamic force. *Inset:* The force distribution when the robot moves towards the left.

S8 Maneuverability of robots under bacterial loading

S8.1 Maximum speed after bacteria assembly

Before loading the bacteria cluster, the micro- or nanorobot can be maneuvered with a very fast velocity. However, it's not possible to trap bacteria with such high moving velocity. As we discussed in S7.3, the hydrodynamic force acting on the bacteria will hinder the thermophoretic trapping force, which will effectively decrease the trapping range. Taking the four motor design as an example, when the laser intensity is 0.3 $mW/\mu m^2$, we can obtain an optical force up to 0.15 pN, which should be balanced with the drag forces acting on the robotic device and trapped bacteria. The total drag force acting on robotic device can be simulated via COMSOL. With a fixed spacing distance, the total drag force should increase linearly with the velocity, as shown in

Fig. S13b. To simplify the model, we assume that the trapped bacteria are spherical with radius r. When the microrobot carries a bacterial cluster, the total hydrodynamic drag force F_{drag} is balanced by the optical thrust generated by the motor design.

$$F_o = F_{drag} = N \times 6\pi\eta r v + Dv \tag{8}$$

where D is the drag coefficient for microrobot extracted from CFD simulations $1.3 \times 10^{-8}\,\mathrm{N/(m/s)}$ in Fig. S13b. N is the effective number of trapped bacteria, η is the viscosity of water, and v is the microrobot velocity. Assuming the robotic device moves with a velocity of $5\,\mu\mathrm{m/s}$ and the average radius of bacteria is roughly 500 nm, then the maximum number of trapped bacteria should be less than $N \leq (F_o/v - D)/(6\pi\eta r) = 2.1$. This also explains that in Supplementary Video 6, the typical moving velocity is quite low. From Fig. S10, we find that, when the moving velocity exceeds 7.5 $\mu m/s$, limited by the drag force, the number of effective trapped bacteria will decrease below one, which indicates that the optical thrust force is not enough to balance the total drag force acting on the robots with trapped bacteria. Another aspect, as discussed in S7.3, the thermophoretic force around the rim of robotic device can reach up to 0.1pN, which is enough to capture the bacteria. This also explains the trapping behavior is most likely around the rim of device.

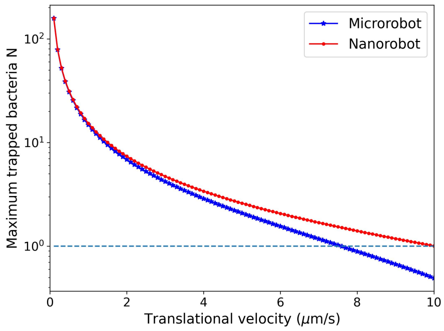

Fig. S10 Maximum trapped bacteria with different translational velocities with a fixed optical thrust force of 0.15pN. The blue dashed line indicates the cutoff velocity which can be used for bacteria trapping.

S8.2 Turning precision after bacteria assembly

The turning behavior is not affected by the presence of trapped bacteria. As shown in Supplementary Video 5, even after the microrobot has assembled a large number of bacteria, its orientation still follows the linear polarization reliably. This is because the bacteria are not rigidly attached to the robot's surface; instead, they remain slightly separated due to electrostatic repulsion via surface charges. Consequently, when the bacterial cluster undergoes random rotational fluctuations driven by Brownian motion, the resulting drag torque does not significantly influence the robot's orientation. The

orientation dynamics remain dominated by the intrinsic optical self-locking mechanism of the microrobot.

S8.3 Cleaning efficiency over a static or mobile robots

In a static trap, in time t, it will mostly clean a single volume of $2\pi R_c^3/3$ around itself. And beyond that, bacteria only reach it by slow Brownian diffusion. In contrast, an actively propelled robot transports this spherical capture volume through space. As it moves with speed v, it effectively sweeps out a much larger "cleaned" volume per unit time, of order:

$$\frac{dV_{mobile}}{dt}/ = (\pi R_c^2 v)/2. \tag{9}$$

Over experimentally relevant timescales, this swept volume exceeds the static capture volume by one to two orders of magnitude, meaning the mobile robot encounters and removes bacteria from many different regions instead of repeatedly interacting with the same local neighborhood. This is consistent with our time-lapse observations, where mobile robots visibly clear extended areas, whereas static hot spots only accumulate bacteria in a confined region.

S8.4 Theoretical estimation of effective Soret Coefficient

To further validate the plausibility of our estimate, we also evaluated the theoretical Soret coefficient of bacteria. Although experimental reports of bacterial S_T are limited, a commonly used expression derived from thermodynamic and electrokinetic considerations [4, 5] is:

$$S_T = -\frac{2\pi R}{k_B T^2}\frac{2\Lambda_l}{2\Lambda_l + \Lambda_p}(\epsilon + T\frac{\partial \epsilon}{\partial T})\zeta^2 \tag{10}$$

Here, R is the bacteria radius, k_B is Boltzmann constant, and Λ_l and Λ_p are the thermal conductivities of the water and bacteria, respectively. The thermal conductivity of bacteria is set to be 0.1 $W/(m \cdot K)$ from the reference [6]. The static permittivity ϵ of water and its temperature derivative $\frac{\partial \epsilon}{\partial T}$ are obtained from the reference [7]. The zeta potential of the solution ζ is taken as -25 mV [8]. Besides, the motion of bacteria close to substrate is significantly influenced by the hydrodynamic boundary effects, which can increase the Soret factor by an enhanced factor of: [9]

$$\Phi(H) = 3(1+H)\frac{(2+6H+3H^2)\ln((H+1)/H) - 1.5(3+2H)}{2+9H+6H^2-6H(1+H^2)\ln((H+1)/H)} \tag{11}$$

where $H = h/R$ and h is the nanoparticle-surface distance.

Figure S11 shows that increasing bacterial size leads to an almost linear increase in the effective S_T, explaining why different bacterial species or cell sizes exhibit different trapping ranges. Our analytical model assumes spherical bacteria, but real cells—especially E. coli—are anisotropic, which introduces additional uncertainty that is difficult to model precisely. Higher surface charge magnitudes (larger $|\zeta|$) also increase S_T, consistent with our observation that S. carnosus—which appears to

carry more surface charge—aggregates more readily and forms more frequent clusters than E. coli. We also note that similar magnitudes of thermophoretic forces have been inferred in related optical-thermophoretic trapping studies. For example, in this paper [10], plasmonic optical fibers were used to assemble E. coli, where thermophoretic forces exceeding ∼100 fN were needed to overcome hydrodynamic drag—comparable to our estimates and consistent with other work [11]. Likewise, another paper reported effective Soret coefficients as large as -3 K^{-1} for Salmonella in dense suspensions, noting that S_T increases steeply at higher concentrations due to frequent cell–cell collisions and long-range hydrodynamic interactions [12].

In our experiments, the effective Soret coefficient is obtained by identifying the equilibrium position where thermophoretic drift balances Brownian motion. This estimate carries inherent uncertainty because different bacterial sizes, shapes, and surface charges produce different trapping radii, and because the assembled clusters are not perfectly symmetric. As reflected in Fig. 4e, cluster diameters vary across experiments due to local intensity variations and biological heterogeneity.

In summary, our reported S_T should be regarded as a reasonable effective estimate, consistent with theoretical expectations and comparable experimental studies. At the same time, it provides a practical approach for rapidly estimating the effective Soret coefficient of bacterial suspensions without requiring complex electrochemical measurements.

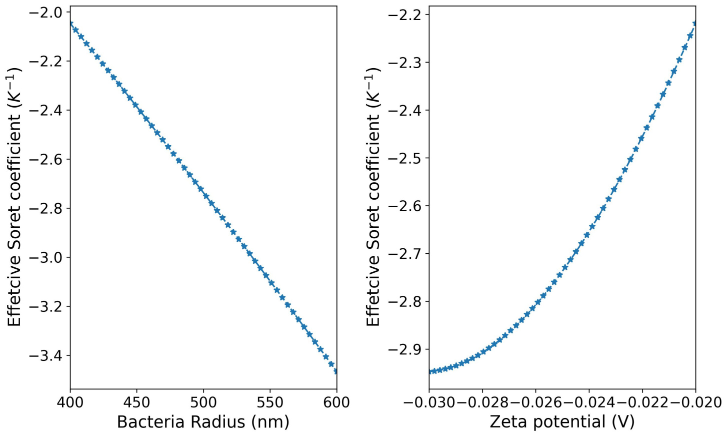

Fig. S11 Calculated effective Soret coefficient based on the theoretical model for (a) varying bacterial radii (with fixed zeta potential $\zeta = -25mV$) and (b) varying zeta potentials (with fixed bacterial radius of 500 nm).

S9 Motion dynamics

S9.1 Out-of-plane stability

In our experiment, the micro- and nanorobot operates in close proximity to the substrate surface (about several hundreds of nanometers), where the downward optical radiation force is balanced by the electrostatic repulsive force from the charged substrate. Taking the microrobot as an example, the out-of-plane optical force F_{oz} was simulated in COMSOL using Maxwell's stress tensor. We found downward optical force F_{oz} = -1.35 pN for a laser intensity of 0.3 $mW/\mu m^2$, mainly originating from optical radiation pressure. The out-of-plane optical force is approximately 7.5 times stronger than the in-plane thrust, providing a strong restoring component that prevents tilting or tumbling even during high-speed motion or turns. Hydrodynamic damping in the viscous medium further suppresses any vertical perturbations. Small out-of-plane velocities are quickly dissipated by viscous drag, ensuring the nanorobot maintains a stable orientation parallel to the substrate.

S9.2 In-plane motion dynamics

The motion dynamics of the micro- and nanorobot can both be described by considering planar Couette flow around an obstacle since the Reynolds number remains very low, even considering that the translational velocity of the nanorobot can reach 50 µm/s. The Reynolds number is estimated as $Re = \rho v L/\mu$, where ρ is the density of water, L is the characteristic length, v is the translational velocity, and μ is the dynamic viscosity of water. Taking the nanorobot as an example, the Reynolds number is approximately 4.6×10^{-5}, indicating a typical laminar flow. The robot remains suspended inside the liquid cell due to surface charges induced by a special surface treatment. When the robot moves within the liquid cell, the drag force balances the optical thrust force apart from short times on the order of XY during which a large acceleration of $a = F/m = 50 m/s^2$ takes place.

Computational fluid dynamics (CFD) simulations based on finite element analysis (FEA, COMSOL) are employed to estimate the translational drag forces and torque near a coverslip interface. The total simulation domain measures $50 \times 50 \times 10$ μm^3. For drag forces simulation, an inlet and an outlet are placed at the yz planes with a background flow velocity u directed along the x-axis, while the remaining exterior planes also have a uniform background velocity u. The boundaries of the nanorobot are set as a no-slip condition. The drag force is computed by integrating the stress along the x-direction over the robot's surface. For the drag-torque simulations, the robot boundary is modeled as a sliding wall with a prescribed angular velocity. The drag torque along the z direction is calculated by integrating the torque density $\tau = yT_x - xT_y$ over the robot surface, where T_x and T_y denote the stress components in the x and y directions.

As shown in Fig. S13a, the simulated drag forces are presented for various spacing distances and translational velocities. With a smaller spacing distance, the device will experience more drag force. Under laminar flow conditions and at a fixed spacing distance, the drag force increases linearly with velocity, as confirmed in Fig. S13b.

From these linear relations, the drag coefficients for both the micro- and nanorobots can be extracted. The microrobot exhibits a larger drag coefficient due to its larger surface area, resulting in greater hydrodynamic resistance during motion in water. Similar behavior is observed for rotational motion, as shown in Fig. S13c, where the drag torque also increases linearly with angular velocity. In the rectangular trajectories shown in Figs. 2c and 3c, we observe that the translational velocity varies with position. In particular, the nanorobot moves faster near the centers of the trajectory sides, where it is closer to the center of the laser spot, and slows down near the corners of the rectangle, where it is slightly farther away. We first rule out contributions from optical gradient forces acting on the device body due to the Gaussian intensity profile of the laser beam. We recalculated this force using full-wave simulations by integrating Maxwell's stress tensor around the robot, as shown in Fig. S12a and b. In Fig. S12a, we present the simulation geometry: the microrobot is propelled in the +y direction by the photon-recoil thrust F_o generated by its plasmonic motors, while the optical gradient force F_{grad}, arising from the non-uniform Gaussian beam, points toward the beam center. Figure S12b shows the position-dependent gradient force obtained by laterally displacing either the microrobot or nanorobot along the x-axis. At an incident intensity of 0.3 $mW/\mu m^2$, the gradient force is below 4 fN, which is two orders of magnitude smaller than the thrust force (∼0.15 pN). This small magnitude is expected given the robots' sub-wavelength dimensions and refractive indices close to that of water.

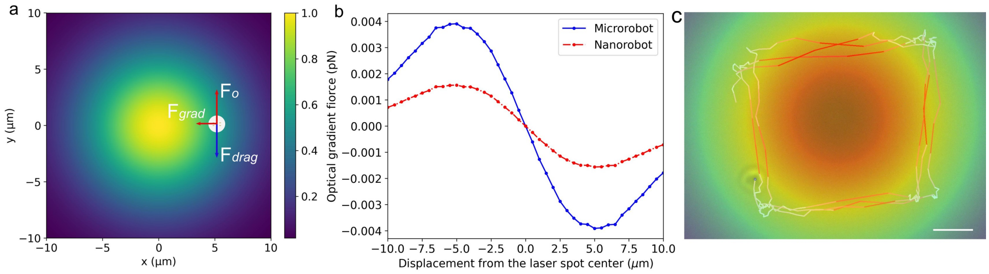

Fig. S12 Simulations for Optical gradient force and position dependent translational velocities. **a**, Simulation geometry. **b**, Position-dependent optical gradient force for micro- and nanorobots. **c**, Overlap between the Gaussian beam profile and measured rectangular trajectory. Scale bar: 2 μm.

Next, we incorporate the position-dependent optical thrust resulting from the Gaussian intensity profile. The local thrust force can be written as:

$$F_o = \alpha I_o e^{(-(2r^2)/w^2} \tag{12}$$

where α is the propulsion force per unit intensity (obtained from Fig. 3b), r is the radial distance from the beam center, and w is the beam waist. Because the optical gradient force is two orders of magnitude smaller, we omit it in the following analysis.

The hydrodynamic drag force F_{drag} is modeled as the product of the drag coefficient D and the translational velocity v. Due to the high acceleration rate of the robotic device, it rapidly reaches a new equilibrium after a change in position, where the drag force balances the position-dependent optical thrust. When the measured rectangular trajectory is overlaid on the two-dimensional Gaussian intensity map (Fig. S12c), regions of high and low velocity correlate well with the local intensity distribution. The instantaneous velocities therefore reflect the local laser intensity and are further influenced by the quality of the Gaussian beam profile.

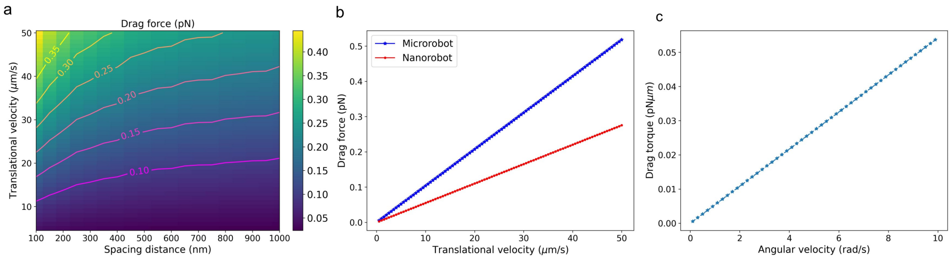

Fig. S13 Simulated drag force and torque. a, Simulated total drag force on the nanorobot as a function of its distance from the glass surface and its translational velocity parallel to the surface. **b**, Linear dependence of the simulated drag force on translational velocity for the micro- and nanorobots (with a fixed spacing distance of 500 nm), from which the corresponding drag coefficients are extracted. **c**, Linear dependence of the simulated drag torque on angular velocity for the microrobot.

S10 Temperature distribution and thermo-induced convective flow

Photo-thermal effects arise from light absorption in plasmonic nanostructures. The resulting temperature distribution can be described by the general heat transfer equation [13–15]:

$$\rho C \frac{\partial T}{\partial t} + \nabla(\kappa \nabla T) = -q(\mathbf{r}) \tag{13}$$

where ρ, C, and κ represent the material density, specific heat capacity, and thermal conductivity, respectively, and $q(\mathbf{r})$ is the heat source density inside the plasmonic structures due to Joule heating. The total heating power is given by integrating the heat density over the entire plasmonic nanostructure: $Q = \int q(\mathbf{r})\, d\mathbf{r} = \sigma_{\text{abs}} I_0$, where σ_{abs} is the absorption cross-section and I_0 is the incident laser intensity. The steady-state temperature distribution for both microrobots and nanorobots immersed in the liquid can be solved using the finite element analysis (FEA) method, as shown in Fig. S10a and c, where the incident laser intensity is the same. As expected, the hottest region is located within the plasmonic structures, which are encapsulated in an HSQ layer. The heat gradually dissipates into the surrounding water environment. An

Table S3 Simulation parameters for heat transfer and laminar flow.

	$\rho(kg/m^3)$	C$(J \cdot kg^{-1} \cdot K^{-1})$	$\kappa(W \cdot m^{-1} \cdot K^{-1})$	$\nu(Pa \cdot s)$
Gold	19300	126	314	\
Water	1000	4184	0.58	1e-3
Silica	2500	703	1.38	\

analytical model based on steady-state heat transfer can be developed by considering a spherical heat source of radius R at the center. Under the consideration of radial symmetry, the heat equation simplifies to:

$$\frac{1}{r^2}\frac{\partial}{\partial r}(r^2\frac{\partial T}{\partial r}) = -\frac{q(r)}{\kappa} \tag{14}$$

Integrating twice, and focusing only on the region outside the heat source $\mathbf{r} > R$, the temperature distribution is given by:

$$\Delta T = \frac{\sigma_{\text{abs}} I_0 R^3}{3\kappa r} \tag{15}$$

Eq. S10 shows that the temperature distribution outside the heating source follows an inverse proportionality with distance, i.e., a $1/\mathbf{r}$ dependence.

Due to the local temperature increase induced by the photothermal effects, the fluid experiences a reduction in mass density, resulting in typical convection flow driven by buoyancy forces. This flow can be described by the Navier-Stokes equation [16–18]:

$$\frac{\partial \mathbf{u}}{\partial t} + (\mathbf{u} \cdot \nabla)\mathbf{u} = -\frac{\nabla P}{\rho} + \nu \nabla^2 \mathbf{u} + \mathbf{F} \tag{16}$$

where $\mathbf{u}$ is the velocity field, P is the pressure, ν is the kinematic viscosity, and $\mathbf{F}$ is the external force. In natural convection, $\nabla P = 0$, and the buoyancy force $\mathbf{F}$ is approximated as:

$$\mathbf{F} = \beta g(T(\mathbf{r}) - T_0)\hat{z} \tag{17}$$

Here, β is the thermal expansion coefficient, g is the gravitational acceleration, $T_0 = 20°C$ is the reference temperature, and $\hat{z}$ is the unit vector in the $+z$-direction.

FEA simulations were used to solve for the convection flow velocity, as shown in Fig. S10b and d for the microrobot and nanorobot, respectively. The simulation parameters can be found in Table. S3. The convection flow is also influenced by the height of the fluid channel, requiring the simulation region to be representative of the actual setup. However, with the same laser intensity applied to both temperature distribution simulations, the convection flow at the relevant plane is extremely small, typically below 2 nm/s. Such a low convection velocity results in a force of only 0.02 fN on a *Carnosus* bacterium with a diameter of 1 µm, which is several orders of magnitude smaller than the thermophoretic force. We agree that such weak flows may help initiate the early-stage motion of nearby bacteria, but they cannot account for the full 3D assembly process. In fact, because convection circulates tangentially around the heated region, it would oppose the trapping of bacteria located above the robot surface. This is consistent with our observation that the assembly occurs throughout a hemispherical trapping volume, which cannot be explained by convection alone.

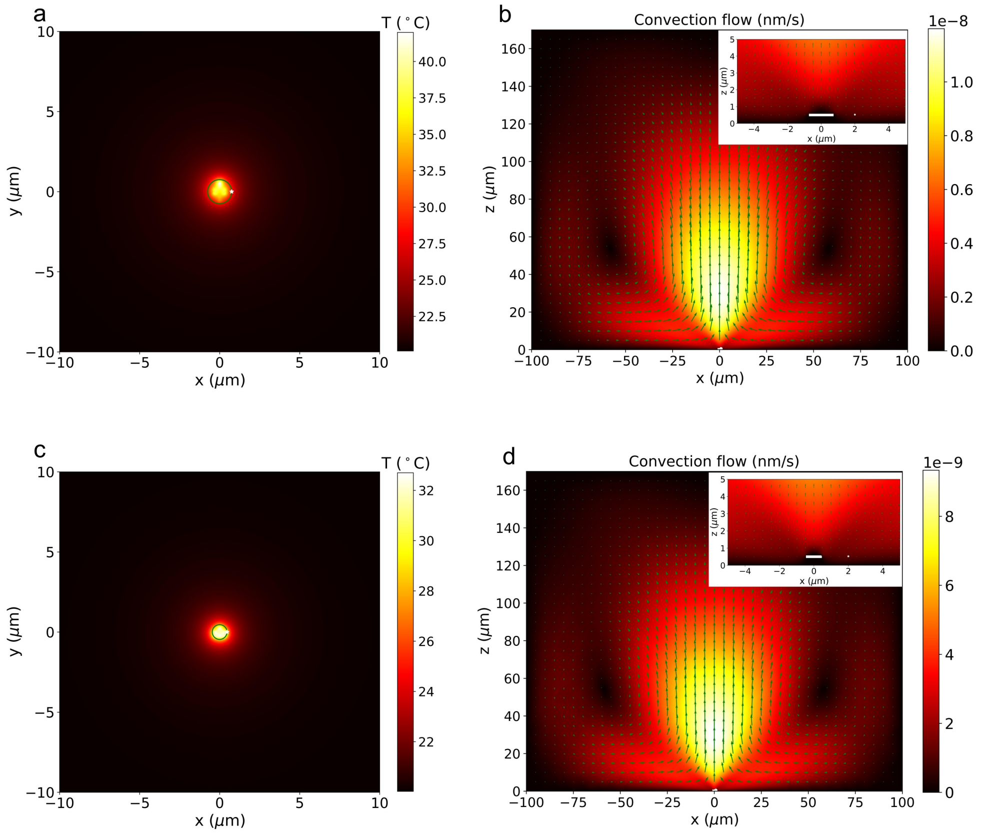

Fig. S14 Temperature distribution and convection flow velocity in micro- (a-b) and nanorobots (c-d). The laser intensity used in the simulations is 0.4 mW/μm^2. In the temperature distribution, the outline of the robots is indicated by a green circle, and a white asterisk marks the position where the temperature is extracted for Fig. 4c. In the convection flow profile, we zoom in on the local region around the robot in the inset, maintaining the same scale bar. The white dot indicates the position where the flow velocity is extracted for Fig. 4c.

References

[1] Simpson, S.H., Benito, D.C., Hanna, S.: Polarization-induced torque in optical traps. Physical Review A—Atomic, Molecular, and Optical Physics **76**(4), 043408 (2007)

[2] Shelton, W.A., Bonin, K.D., Walker, T.G.: Nonlinear motion of optically torqued nanorods. Physical Review E—Statistical, Nonlinear, and Soft Matter Physics **71**(3), 036204 (2005)

[3] Righini, M., Ghenuche, P., Cherukulappurath, S., Myroshnychenko, V., Abajo,

F.J., Quidant, R.: Nano-optical trapping of rayleigh particles and escherichia coli bacteria with resonant optical antennas. Nano letters **9**(10), 3387–3391 (2009)

[4] Anderson, J.L.: Colloid transport by interfacial forces. Annual review of fluid mechanics **21**(1), 61–99 (1989)

[5] Putnam, S.A., Cahill, D.G., Wong, G.C.: Temperature dependence of thermodiffusion in aqueous suspensions of charged nanoparticles. Langmuir **23**(18), 9221–9228 (2007)

[6] Inomata, N., Miyamoto, T., Okabe, K., Ono, T.: Measurement of cellular thermal properties and their temperature dependence based on frequency spectra via an on-chip-integrated microthermistor. Lab on a Chip **23**(10), 2411–2420 (2023)

[7] Archer, D.G., Wang, P.: The dielectric constant of water and debye-hückel limiting law slopes. Journal of physical and chemical reference data **19**(2), 371–411 (1990)

[8] Liang, X., Liao, C., Thompson, M.L., Soupir, M.L., Jarboe, L.R., Dixon, P.M.: E. coli surface properties differ between stream water and sediment environments. Frontiers in Microbiology **7**, 1732 (2016)

[9] Würger, A.: Hydrodynamic boundary effects on thermophoresis of confined colloids. Physical review letters **116**(13), 138302 (2016)

[10] Kim, J.A., Yeatman, E.M., Thompson, A.J.: Plasmonic optical fiber for bacteria manipulation—characterization and visualization of accumulation behavior under plasmo-thermal trapping. Biomedical Optics Express **12**(7), 3917–3933 (2021)

[11] Chen, J., Cong, H., Loo, J., Kang, Z., Tang, M., Zhang, H., Wu, S.-Y., Kong, S.-K., Ho, H.-P.: Thermal gradient induced tweezers for the manipulation of particles and cells. Scientific reports **6**(1), 35814 (2016)

[12] Kai, T., Abe, T., Yoshinaga, N., Nakamura, S., Kudo, S., Toyabe, S.: Collective gradient sensing by dilute swimming bacteria without clustering. Physical Review Research **6**(3), 032061 (2024)

[13] Govorov, A.O., Richardson, H.H.: Generating heat with metal nanoparticles. Nano today **2**(1), 30–38 (2007)

[14] Baffou, G., Quidant, R., Abajo, F.J.: Nanoscale control of optical heating in complex plasmonic systems. ACS nano **4**(2), 709–716 (2010)

[15] Jauffred, L., Samadi, A., Klingberg, H., Bendix, P.M., Oddershede, L.B.: Plasmonic heating of nanostructures. Chemical reviews **119**(13), 8087–8130 (2019)

[16] Guyon, E., Hulin, J.P., Petit, L., Mitescu, C.D.: Physical Hydrodynamics. Oxford University Press, Oxford (2015)

[17] Donner, J.S., Baffou, G., McCloskey, D., Quidant, R.: Plasmon-assisted optofluidics. Acs nano **5**(7), 5457–5462 (2011)

[18] Roxworthy, B.J., Bhuiya, A.M., Vanka, S.P., Toussaint Jr, K.C.: Understanding and controlling plasmon-induced convection. Nature communications **5**(1), 3173 (2014)
