# Supplementary material for: A nanoscale robotic cleaner
Source: Nat Commun. 2026 Mar 27;17:3027. doi: 10.1038/s41467-026-70685-9 (PMC13035946; doi:10.1038/s41467-026-70685-9)
Supplement: Supplementary file 2 — Description of Additional Supplementary Information [file 41467_2026_70685_MOESM2_ESM.pdf]

Description of Additional Supplementary Files

File Name: Supplementary Video 1
Description: Steering a microrobot along a rectangular trajectory using varying laser intensities.

File Name: Supplementary Video 2
Description: Navigating a nanorobot along a rectangular trajectory using varying laser intensities.

File Name: Supplementary Video 3
Description: Examples of a nanorobot propulsion along the controlled trajectories of "E", "P" and "5".

File Name: Supplementary Video 4
Description: An example of a nanorobot propulsion along spiral-shaped rectangular trajectories.

File Name: Supplementary Video 5
Description: An example of steering a microrobot capture, transport, and reversibly assemble different bacteria (*Escherichia coli* and *Staphylococcus carnosus*) in suspension.

File Name: Supplementary Video 6
Description: Examples of propelling a nanorobot loaded with multiple bacteria along controlled trajectories.

File Name: Supplementary Video 7
Description: A demonstration of microrobotic cleaner. The blue dashed circle indicates the size of laser spot.

File Name: Supplementary Video 8
Description: A demonstration of nanorobotic cleaner. The blue dashed circle indicates the size of laser spot.
